# Ondansetron-related hemorrhagic posterior reversible encephalopathy syndrome (PRES) following gastric bypass

**DOI:** 10.1186/s40064-015-1644-9

**Published:** 2016-01-06

**Authors:** M. Alain Babi, Mark J. Gorman, Marilyn J. Cipolla, Gilman Allen, Salman Al Jerdi, Ryan Clouser, Christopher Commichau

**Affiliations:** Department of Neurological Sciences, The University of Vermont Medical Center and the University of Vermont College of Medicine, Burlington, VT 05405 USA; Department of Medicine, Division of Pulmonary Medicine and Critical Care, The University of Vermont Medical Center and the University of Vermont College of Medicine, Burlington, VT 05405 USA; Division of Neuro-critical care, Department of Neurology, Duke University Hospital, DUMC 2900, Durham, NC 27710 USA

**Keywords:** Stroke, Intracerebral hemorrhage, Cerebral autoregulation, Imaging, Intracranial hypertension, Intracranial pressure, Ondansetron, PRES (posterior reversible encephalopathy syndrome)

## Abstract

**Background:**

Posterior reversible encephalopathy syndrome (PRES) is a clinical-radiographic syndrome formally recognized in 1996, which describes specific changes noted on neuroimaging thought to be related to impaired cerebral blood flow autoregulation and endothelial dysfunction. We report a case of PRES in the setting of increased ingestion of ondansetron; complicated by hemorrhagic transformation and refractory intracranial hypertension. We hypothesize an association of 5-HT_3_ antagonism and PRES.

**Findings:**

This is a case study report; with review of previously published literature through PubMed search. We describe the case of a 25 year old man following bariatric surgery who increased his ingestion of ondansetron, taking up to 40 tablets/day due to excessive nausea and vomiting. The patient was hospitalized for progressively more severe headache of 1 week’s duration. Computed tomography (CT) revealed bilateral cerebral edema in the parietal and occipital lobes in the setting of elevated blood pressure (BP). Three days into his admission, following improvement in his BP with oral anti-hypertensive but continued use of the ondansetron, the patient developed near complete blindness. CT head imaging revealed progression of the posterior cerebral edema and intraparenchymal hemorrhage. He was admitted to our ICU and despite supportive treatment, his neurological examination worsened while CT head imaging findings remained stable. Invasive multimodality monitoring revealed elevated intracranial pressure. The patient was aggressively treated and after a prolonged hospitalization and rehabilitation course, made a significant recovery.

**Conclusion:**

This case highlights a very rare potential neurological complication of ondansetron, a commonly used medication. We hypothesize an underlying association between PRES and 5-HT_3_ antagonism, via the latter’s potential role in endothelial dysfunction. Prompt recognition and treatment of PRES is essential, in order to prevent secondary cerebral injury and the associated potentially grave consequences.

## Background

PRES is a clinical-radiographic syndrome of uncertain pathogenesis but of multiple heterogeneous etiologies that are commonly grouped together because of similar radiographic findings. Described using varying names in different case reports, the term reversible posterior leukoencephalopathy syndrome (RPLS) was first coined in 1996 by Hinchey et al. (1996). While the newly-coined term RPLS incorporated the same clinical and radiographic findings of what was then known as the hypertensive encephalopathy syndrome (Stott et al. 2005; Schwartz et al. 1992; Schwartz et al. 1995; Hauser et al. 1988). An extensive body of literature of this syndrome has since accumulated; and the prior term “RPLS” has been recognized as misleading as well. The reasons being is that many cases can be irreversible or may even be fatal, and neuroimaging lesions may not be restricted to the white matter or posterior cortex as the name suggests (Hinchey et al. 1996; Stott et al. 2005; Staykov and Schwab 2012; Covarrubias et al. 2002).

The pathogenesis of PRES remains unknown; however, several hypotheses have been suggested. PRES appears to be related to impaired cerebral blood flow autoregulation, as well endothelial dysfunction (Hinchey et al. 1996; Stott et al. 2005). Wide varieties of medical conditions and medications have also been implicated (Hinchey et al. 1996; Stott et al. 2005; Strandgaard and Paulson 1984; Staykov and Schwab 2012). Autoregulatory failure, reactive focal vasoconstriction resulting in local hypoperfusion, cytotoxic cerebral edema, and cerebral infarction have been reported as well (Hinchey et al. 1996; Stott et al. 2005; Strandgaard and Paulson 1984; Staykov and Schwab 2012; Hefzy et al. 2009; Covarrubias et al. 2002; Golombeck et al. 2013). Endothelial dysfunction has been implicated, particularly in PRES associated with pre-eclampsia, immunosuppressive and cytotoxic therapies (Hinchey et al. 1996; Stott et al. 2005; Staykov and Schwab 2012). The latter may lead to direct cerebral endothelial dysfunction and toxicity leading to blood–brain barrier disruption and capillary leakage which may trigger further vasogenic edema (Stott et al. 2005; Strandgaard and Paulson 1984; Staykov and Schwab 2012). The clinical manifestations of PRES are varied and may range from insidious onset of headache, confusion, seizure, visual changes, or decreased level of consciousness to deep coma (Hinchey et al. 1996; Stott et al. 2005). Mortality directly related to PRES has been reported (Hinchey et al. 1996; Alhilali et al. 2014; Golombeck et al. 2013). Treatment of PRES remains supportive with control of blood pressure in hypertensive patients and removal of offending agents where identified.

In this report, we describe a case of PRES complicated by intracerebral hemorrhage in the setting of excessive ingestion of ondansetron following bariatric surgery. We hypothesize an association of 5-HT_3_ antagonism and PRES, implicating PRES as a rare potential complication of a commonly used medication.

## Case report

**Fig. 1:**
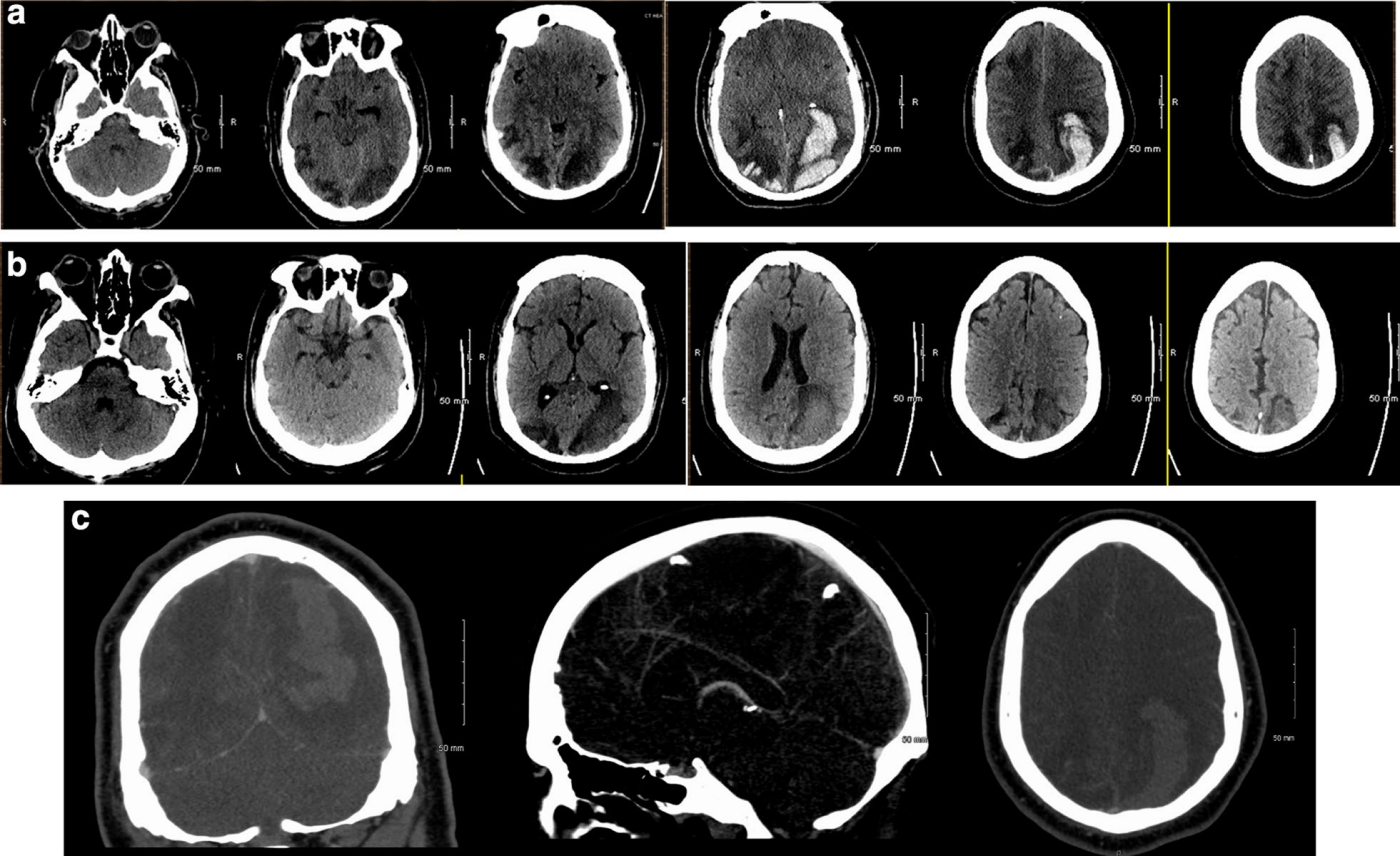
**a** Non-contrast CT head *(left to right: caudal to rostral)* demonstrating multifocal (particularly in the posterior circulation) cerebral edema with associated intracerebral hemorrhage, mostly centered on left > right occipital lobes and extending into the fronto-temporal region. **b** Non-contrast CT follow-up at 12 weeks in same sequence. **c** CT venography (*left*: coronal, *middle*: sagittal, *right*: axial) demonstrating patency of visualized venous system; and without any evidence of venous obstructions

**Fig. 2:**
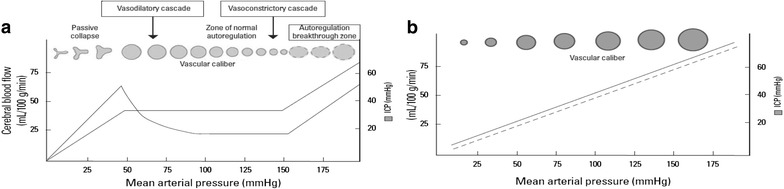
**a** Cerebral autoregulatory curve under normal physiological condition. **b** Cerebral autoregulatory curve under impaired situation. (With permissions, courtesy of KO SB, J Stroke. 2013;15(2):99–108)

A 25-year-old morbidly obese male underwent bariatric surgery 3 months prior to presentation. In the week preceding his presentation to an outside hospital, he had increased his intake of ondansetron 4 mg tablets up to 40 tablets/day because of intractable nausea and vomiting. He was initially hospitalized for a progressively worsening headache of 1 week duration. Initial computed tomography (CT) revealed bilateral cerebral edema in the parietal and occipital lobes in the setting of elevated blood pressure (BP). However, 3 days into his admission, following initial improvement in his blood pressure with oral anti-hypertensive medication, but in the setting of continued use of the ondansetron, he developed sudden onset near complete blindness with inability to count fingers from one foot away but bare light perception. His blood pressure was measured as 190/110. Computed tomography (CT) head imaging revealed similar cerebral edema in the posterior cortex but new intra-parenchymal hemorrhage (Fig. 1). The patient was transferred to the intensive care unit (ICU) of our institution but despite supportive treatment, his condition worsened while CT imaging findings remained unchanged. No angiography was obtained, as it was felt to likely be inconclusive in the setting of massive hemorrhage. A CT venogram demonstrated no evidence of venous sinus thrombosis. Invasive intracranial monitoring with a Licox^®^ monitor revealed variable to elevated intracranial pressure (ICP) ranging from 10 to 50 mm Hg. The patient was aggressively treated with hyperosmolar therapies and other supportive measures. He stabilized and after a protracted hospitalization was eventually discharged to rehabilitation. At the 12 week follow-up, he was noted to be independent in most of his activities of daily living. He was, however, left with significant visual impairment, leaving him only with the ability to count fingers in central vision, and only detect movement in his right visual fields. Follow-up CT of the head without contrast was obtained at that time, (Fig. 2) revealing resolution of the hematomas.

## Discussion

This case highlights a rare potential neurological complication of ondansetron; that of PRES complicated by intracerebral hemorrhage and refractory intracranial hypertension.

The details of PRES pathogenesis are not entirely clear, but the condition appears to be related to impaired cerebral autoregulation and endothelial dysfunction. The association of PRES with diverse medications and clinical entities could either suggest a unified pathogenesis or differing mechanisms yielding similar clinic-radiographic pictures. The predilection for primary involvement of the posterior brain regions is not well understood either. Several theories have been postulated; one thought to involve regional heterogeneity in the sympathetic innervation of intracranial arterioles that serve to protect the brain during episodes of severe hypertension (Edvinsson et al. 1976). In support of this, histochemical staining has demonstrated a greater concentration of adrenergic nerves around pial and intracerebral vessels in the anterior circulation compared to the posterior circulation (Beausang-Linder and Bill 1981; Tecott et al. 1993).

Ondansetron is a highly specific and selective serotonin 5-HT_3_ receptor antagonist with low affinity for dopamine receptors (Christofaki and Papaioannou 2014). 5-HT_3_ receptors are widely expressed in the intracranial pial vasculature (Hefzy et al. 2009; Christofaki and Papaioannou 2014; Tecott et al. 1993; Kou et al. 2002; Bétry et al. 2015). The mechanism through which ondansetron might contribute to the development of PRES may relate to its effect on blood vessels (Hefzy et al. 2009; Christofaki and Papaioannou 2014), as prolonged antagonism of these receptors (from the consistent and massive ondansetron ingestion by our patient) at the micro- and macro-vascular level may ultimately have had a toxic effect on the vascular endothelium, leading to capillary leakage and blood–brain barrier disruption, and ultimately progressive vasogenic cerebral edema. It is also worth noting that many cases of PRES associated with cytotoxic or immunosuppressive drugs (such as severolimus, cyclosporine, tacrolimus, bevacizumab, cisplatin) have been reported in normotensive individuals, and in the setting of non-toxic levels or doses of these drugs, implying a direct cytotoxic-effect on the intracranial vasculature. Eight cases of ondansetron-related PRES have been reported to the FDA between January 2004 and October 2012 ( 2015), though detailed information about these is unavailable.

We hypothesize that elevated blood pressure may have been an inciting event, which when coupled with ingestion of ondansetron culminated in the development of PRES by means of pharmacologically amplified endothelial dysfunction and a resultant disruption of the brain–blood barrier (Hinchey et al. 1996; Stott et al. 2005; Glusker et al. 2006; Covarrubias et al. 2002; Ozkan et al. 2014; Schwartz et al. 1995). In this particular patient, the finding of progressive hemorrhagic PRES despite optimized control of our patient’s blood pressure, and the subsequent elevation in his ICP with otherwise unchanged imaging findings suggest that ondansetron led to uncontrolled vasogenic edema from endothelial dysfunction and possibly impaired cerebral autoregulation.

Hemorrhage in PRES has also been reported by others. For example, Hefzy et al. reported incidence of hemorrhage with PRES in 15.2 % of the case series, with the highest rate of hemorrhage observed in the setting of immunosuppressive therapies (22 %) and the lowest rate in patients with eclampsia (5.5 %) (Hefzy et al. 2009). This higher incidence of hemorrhagic PRES in the setting of concurrent immunosuppressive or cytotoxic drugs may relate to a direct cytotoxic effect on the cerebral microcirculation and blood brain barrier.

PRES may lead to permanent injury or death. Historically, PRES associated with hemorrhage has been reported to be associated with 16–29 % mortality rate (Akins et al. 2014) whereas most recent reports indicate 3–6 % mortality during a range of follow-up time (generally 1–3 months) (Akins et al. 2014; Fugate and Rabinstein 2015; Moon et al. 2013). Severe neurological injury and fatality in PRES have most often occurred in relation to intracranial hemorrhage, posterior fossa edema with brainstem compression or herniation, or diffuse cerebral edema and increased intracranial pressure (Fugate and Rabinstein 2015). Some long-term recognized sequelae of PRES include persistent hemiparesis, seizures, impaired vision, and decreased cognition (Fugate and Rabinstein 2015). Additionally, several studies have reported that incomplete recovery in PRES is often associated with intracerebral hemorrhage (Fugate and Rabinstein 2015; Moon et al. 2013; Shaharir et al. 2013).

We recognize several limitations to this case report. The inability in further obtaining any further information regarding the eight prior cases of ondansetron-related PRES (safety reports of the FDA) adds to the limitations of this case report. The absence of cerebral angiography restricts any potential insight towards an underlying vascular abnormality that may have contributed to this presentation albeit highly unlikely given lack of clear clinical stigmata during subsequent follow-up routine CT head. Additionally, it is difficult to completely exclude that uncontrolled hypertension was the cause in this case, although the severity of the symptoms, their refractoriness to anti-hypertensive therapy and the association with prolonged high-dose ingestion of ondansetron appears to implicate this drug as at least a potentially contributing agent.

## Conclusion

PRES is a clinico-radiographic syndrome of heterogeneous etiologies that are poorly understood, but grouped together because of similar neuro-imaging findings. Different etiologies have been implicated, with uncontrolled blood pressure elevation as the most frequent culprit. Furthermore, systemic medications and other cytotoxic or immunosuppressive medication can contribute or solely lead to this condition by separate mechanisms. Prompt recognition and treatment of the precipitating condition and prevention of secondary insults such as intracranial hemorrhage, intracranial hypertension, and cerebral infarction are essential.

We believe physicians need to have greater awareness of this potential complication of a routinely used medication. In this manuscript, we report an observation of ondansetron overdosage associated with hypertension leading to PRES with potentially disastrous consequences. We propose a possible causative mechanism, mainly focused on ondansetron’s anti-serotonergic properties and their relationship with cerebral autoregulation.
